# Multidimensional relative poverty of rural women: Measurement, dynamics, and influencing factors in China

**DOI:** 10.3389/fpsyg.2022.1024760

**Published:** 2022-10-13

**Authors:** Yan Peng

**Affiliations:** Business School, Huang Gang Normal University, Huang Gang, China

**Keywords:** multidimensional poverty, rural women, measurement, dynamics, influencing factors, evidence from the Chinese family panel survey

## Abstract

**Background:**

China has eliminated absolute poverty; however, relative poverty still exists. Specifically, the characteristic group of rural women is affected by traditional gender concepts and behavioral norms, and rural women are often relatively deprived of economics, rights, abilities, and information.

**Objective:**

Therefore, studying their relative poverty is crucial for realizing the overall development of China and the shared prosperity of farmers.

**Methods:**

This study utilizes the multidimensional poverty theory to construct an analytical framework for the multidimensional relative poverty of rural households using the Alkire-Foster multidimensional measurement method and the tracking survey data of the “China Family Tracking Survey” from 2010 to 2016. From the “individual perspective,” the six dimensions of economy, health, humanities, spiritual life, social relations, and rights are used to measure and compare the relative poverty of women and men in rural households in China.

**Results:**

The results showed that the relative poverty of women in rural households is extensive and broader, especially in the economic, humanities, and rights dimensions, and is much higher than that of men. Education level, physical health, ideology, and family status are the primary factors affecting the multidimensional relative poverty of women.

**Conclusion:**

This study finds that the relative poverty of rural women exists within the family and it is multi-dimensional. This finding provides a reference for promoting well-rounded human development and achieving common prosperity for everyone.

## Introduction

Developing countries have significant regional and gender differences in poverty (Finnoff, 2015). The incidence rate for female poverty is much higher than that of men, and the situation is worsening (Jinzi, 2014). United Nations research found that women are more likely to be poor (United Nations, 2015). International Fund for Agricultural Development also observed that since the 1980s, women’s rural poverty growth rate has increased significantly faster at 48% compared to 30% for men worldwide (Chao, 2019). China also faces the phenomenon of women’s impoverishment, and women are often relatively deprived of economics, rights, abilities, and information (Yanping 2021; Jing and Chenghu, 2022).

To eliminate women’s poverty and promote women’s development, China formulated three “Programs for the Development of Chinese Women” in 1995, 2001, and 2011, respectively. Additionally, to implement poverty alleviation and development work, local governments actively performed the “Women’s Poverty Alleviation Action” and created various public welfare brands to support women in poverty-stricken areas. China has made remarkable achievements in promoting poverty alleviation, and the problem of absolute poverty among women has significantly improved. However, the traditional gender norms and the gender division of labor in families put rural women at a disadvantage for possessing living assets and resources and in the decision-making of family and production activities. If rural women lose family support, their poverty concerns become prominent, and the existing policies, such as industrial poverty alleviation, have a limited effect on women-led families. This is often ignored due to its concealment within the family. Their economic decision-making power and cultural, educational, and social participation rights are easily obscured (Deping and Kai, 2020). The precision work mechanism has achieved the coverage and precision of “households” (Ling, 2018); however, the individual poverty concern remains unresolved, not conducive to rural development and the shared prosperity of farmers.

Theoretical research and poverty alleviation practice in China previously did not focus on gender differences. Identification, breadth, depth, long-term solution, and vulnerability of multidimensional poverty based on “households” have always been the focus of research. This may be because the traditional research method assumes that resources are evenly distributed within the family, family members share, and there is no gender difference in poverty. Scholars have now gradually introduced a gender perspective into the poverty eradication concern to distinguish the different manifestations and impacts of poverty among women and men. The research primarily involves women’s poverty definition (Valentin, 2000; Xiaoyun and Qiang, 2006), types and characteristics (Shengju, 2016; Wenfeng, 2018), and causes. Studies show that female poverty is primarily affected by gender attitudes, education level, income level, social system, and social structure (Brucker et al., 2014; Xiaoyun and Xiaoyun, 2014). Additionally Guangyan (2016), Xiaoying and Hexia (2016), Yun (2018), and Jianping (2018) measured and decomposed the multidimensional poverty of women and observed that women’s poverty is in terms of economy, and also personal ability, and welfare. However, few studies have used multi-dimensional poverty theories and methods to study gender differences in poverty, including Haitao and Yu (2013), Jianping and Mimi (2018), and Jiquan (2022). Moreover, few comparative studies exist on multidimensional dynamic poverty in women and men using the Alkire-foster method. Studies have mainly selected indicators from the “family perspective” to research poverty. Measurements cannot scientifically and comprehensively examine the multidimensional poverty status of a single individual in rural households.

Therefore, this study uses the tracking data of the “China Family Tracking Survey” from 2010 to 2016, following the individual characteristics of the rural family population from the “individual perspective,” with six dimensions of economy, health, humanities, spiritual life, social relations, rights, and corresponding indicators to compare and analyze the multidimensional relative poverty among women and men in rural households and its changes. Further, it uses the ordered Probit model to analyze the factors influencing the multidimensional relative poverty of women in rural households from three levels of female individual, family, and village-level characteristics. This study is novel because it measures gender differences in multidimensional relative poverty, and we attempt to provide recommendations for the overall development of rural women and the common prosperity for everyone.

## Literature review

Women’s poverty in rural families has several causes, and its formation is comprehensively affected by the cultural system, society, family, and individuals. Relevant scholars have conducted research on the causes of women’s poverty from different perspectives, primarily focusing on the following aspects.

### The mechanism of rural women’s poverty from an economic perspective

Income level is a direct factor that measures individual poverty. Due to the influence and restriction of the gender division of labor, women must focus more on family affairs and are expected to choose less rewarding occupations to care for the family, resulting in their corresponding less effort in work and income return (Xiaojing, 2018). The mechanism of women’s income level on their poverty is primarily reflected in two aspects: (1) Direct effects: Once rural families fall into poverty, or women in the family lose their economic dependence on men, they will inevitably fall into economic poverty; (2) Indirect effects: Women’s income disadvantage weakens their ability to negotiate in the allocation of family resources. This leads to gender differences between women and men in the household regarding ownership of living assets, resources, physical distribution, health expenditures, and personal consumption.

### The mechanism of rural women’s poverty from a social perspective

#### Human and social capital

Women’s comparative disadvantage in the division of labor, economic efficiency reasons such as childbirth and related costs, and non-economic efficiency factors such as traditional cultural customs, gender role identification, and other non-economic efficiency factors have led to the deep-rooted belief that the return on investment in women is lower than men (Weihua, 2015), leading to reduced investment in females at the family and societal level. Rural families have the traditional concept of “raising children to support the elderly” and the expected return of investment in women. When family resources are limited, men are prioritized for the family’s nutrition distribution, education, and social service utilization of various resources (Hua and Xiaogang, 2011; Hong and Zhihong, 2014). The limited resources, lack of education access, and training and production resources for rural women compared to men hinder the effective development of their individual human resources (Junwen, 2013).

Social capital plays a positive role in anti-poverty (Hengyan and Longbao, 2013; Qingzheng and Tianlun, 2014); however, it is not balanced, specifically regarding gender differences (Min, 2008). Women are weaker than men in the foundation, space, and ability to mobilize resources. Hence, they lack social capital compared to men (Qianyun, 2007), which is also why women are vulnerable to poverty.

#### Social exclusion

Social exclusion refers to that of the marginalized poor and weak groups by the dominant group at different levels of social consciousness and policies and regulations. The poverty of women, older adults, and other groups is essentially caused by social exclusion (Renwei, 2018). The labor market regards the cost of women’s childbirth and raising children as a burden affecting labor efficiency and often shows direct or subtle gender discrimination against women, causing disadvantages in the labor market for women. The prejudiced gender role identification in society leads to a lack of opportunities for women to gain power, education, training, and development (Xiaoyun et al., 2004). The long-term lack of social participation, social exclusion, and deprivation of power worsen women’s poverty during old age.

#### Social security policy

Due to the traditional dual structure system of urban–rural division, it is challenging for farmers to gain equal economic, political, social, and cultural rights as urban residents, leading to poverty (Dengwen and Jia, 2014). The relief effect of the rural social security system is obvious, reducing the depth and intensity of poverty in rural areas (Jie, 2012). The rural subsistence allowance system’s substantial assistance and diet guarantees have effectively ensured the rights of subsistence allowance households, the old-age security system with economic welfare characteristics has effectively alleviated expenditure and income poverty, and the medical security system has effectively weakened the vicious circle of “poverty” and “disease” (Yafang, 2014; Wanting, 2015; Yiwei, 2018). However, the current social security policy in rural areas lacks the need for rural social development and a gender-sensitive perspective in its establishment and improvement.

Most women are in informal employment and farming, and their resilience is insufficient to resist natural and social risk shocks (Yunxiang, 2017). Hence, women’s needs for social security should be higher than men’s. However, the social security system based on a patriarchal society inevitably has gender inequality (Wenli, 2005). Women from rural households often spend more time on unpaid work. As informal workers, they are at a disadvantage in the social security system (Jing and Wenhui, 2014). Additionally, when women from families dominantly performing unpaid care work become older, it is challenging for public social policies to provide them with sufficient economic security, further causing gender stratification in the social security system (Manxiu and Cailong, 2015).

#### Intergenerational transmission of poverty

The intergenerational poverty concern has been studied since the 1950s. The “Blauer-Duncan Model” provides a quantifiable analysis framework for the intergenerational transmission of poverty. Children from low-income families are at a disadvantage for education, employment, and health, leading to the inheritance and replication of poverty and its disadvantages between generations, thus forming the intergenerational transmission of poverty (Mingang and Ruili, 2012). Most impoverished children of families from poverty did not substantially improve their income status after evading poverty, and there is still a greater possibility of them returning to poverty (Lidong, 2013; Jianhua, 2016). As the family’s primary caregiver, particularity in women’s role in raising offspring and the future development of the family makes it possible for them to pass on the conditions and factors leading to poverty to their children (Aijun, 2009; Chun and Yuanfu, 2011). Shuguo (2018) described the intergenerational transmission mode of intellectual poverty between mothers and children; in the “low education level-low wealth capital-low social capital,” the next generation falls in a vicious relationship between these, forming a “dead knot.”

### The mechanism of rural women’s poverty from the institution perspective

The traditional preference for boys over girls in rural China, coupled with the mandatory family planning measures in the 1980s, has led to the lack of survival rights for women and children in rural families (Puwan and Huiyong, 2010) and has led to physical and psychological pressure caused by constant childbirth in women and the breakdown of marital relations due to fertility restriction challenges. Additionally, the lack of protection system resources provided by laws and regulations for women’s equal rights, the prevailing marriage customs in rural areas, the disregard and deprivation of women’s property rights, and women in rural families losing their land and homesteads without compensation (Zhibin, 2005; Qun and Yunxian, 2011) makes them more likely to fall into poverty. Furthermore, policies in periods of social transformation, industrial upgrading, and structural adjustment often have gender blind spots, and there is a lack of gender assessment of policy adjustments (Yihong, 2016).

### The mechanism of rural women’s poverty from the cultural perspective

“Because the poor have lived in poverty for a long time, they have formed specific lifestyles, behavioral norms, and values, and this lifestyle, behavioral norms, and values will be passed on from generation to generation, forming a unique culture of poverty” (Lewis, 1959). Rural women’s cultural poverty is a backward state wherein female individuals lag behind contemporary economic and social development for education, individual subjectivity, individual perception, values, behavior patterns, and lifestyles, affecting their survival and development (Jinzi, 2014). Gender is formed based on social culture construction, and the traditional concept of gender is a crucial incentive for women’s poverty. The old gender division of labor and historical traditions put women in the shackles of gender stereotypes, restricting or hindering women’s access to education, development potential, and improving personal qualities (Qinqin, 2018). Women conditioned to poverty culture willingly play the role of traditional family women. The lack of cultural knowledge and the indifference to modern social concepts and thoughts confine them to an impoverished outlook on life. They dare and do not want to break their life status quo.

### Constructing a multidimensional poverty framework for rural women

Deriving on the research on the formation mechanism of rural women’s relative poverty, women’s relative poverty group is easily concealed, and the unfair distribution of resources and rights affects women’s rights, opportunities, and achievements throughout their lives. Hence, women’s impoverishment has multiple manifestations. They are more likely to be relatively deprived of the economy, rights, health, ability, and information, resulting in their relative poverty.

**Figure 1:**
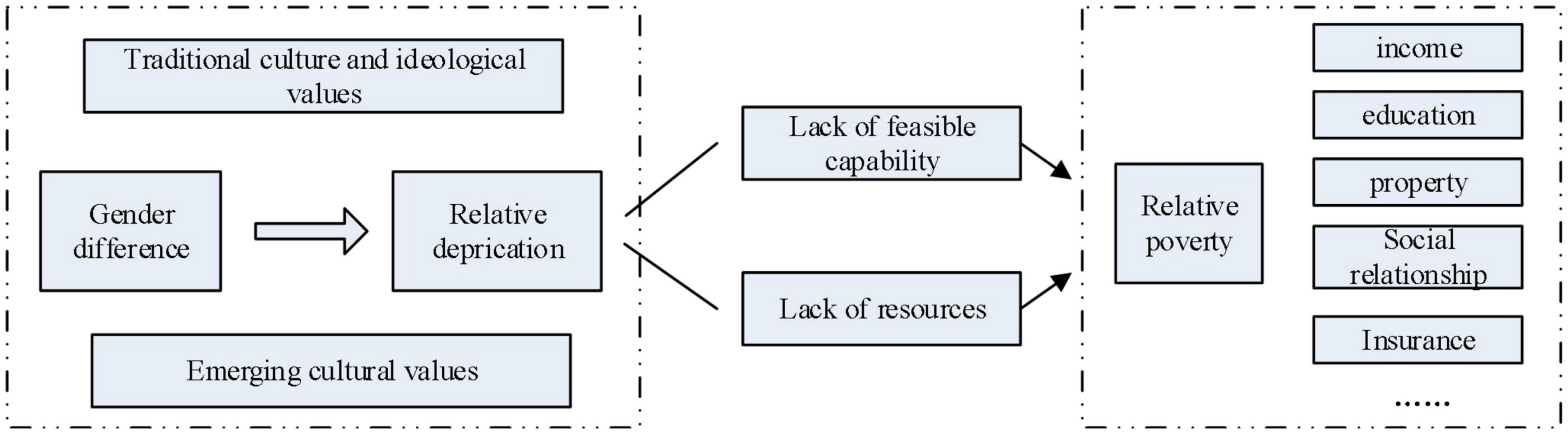
The theoretical analysis framework of rural women’s relative poverty.

This study used the mindsponge theory to explain the multidimensional situation of women’s relative poverty, as shown in Figure 1.

Is the formation of rural women’s relative poverty a complex process of multi-dimensional overlapping? What dimensions of relative poverty are more likely to affect rural women? We can answer this question using several principles proposed by the mindsponge mechanism, “a framework that explains how an individual absorbs and integrates new cultural values into their own set of core values and the reverse of ejecting waning ones” (Vuong and Napier, 2015, pp: 359).

Chinese Confucian culture has a long history. Concepts like “three obedience and four virtues,” “male superiority,” “patriarchal power,” and “husband power” confine women to a fixed development model. Women’s education level, family marriage, social status, spiritual and cultural needs, and public participation have been wanting for a long time. Based on this, the outside world uses norms, values, and other criteria to restrict and judge women’s roles, the manner of performing them, and their outcome. Women and their families assimilate and recognize these values and beliefs to some extent.

There is a gender difference in roles caused by the preference for boys over girls, and rural households generally believe that investing in boys is more rewarding, which means girls’ opportunities for education and investment in education are often lower than the boys’. This leads to educational poverty.

At the same time, with the emergence of some new cultural values, such as the “Protection of the Rights and Interests of Married Women,” some women have realized that their rights and interests have been treated unfairly, but the deep-rooted village rules and regulations have continued to prevent them from accessing their rights and deprived them of their interests. For example, in the practice of distribution of land rights and interests in China, women may be identified as “outsiders” in both their parents’ and in-laws’ homes and cannot enjoy the rights and interests prevalent in their village. Even though men and women are considered equal by law, the rights and interests of women in rural areas are often limited and violated by village rules and regulations. This creates a poverty of rights.

Under traditional gender norms and concepts, rural families mainly have a male-centered structure, and role division is obvious. Male migrant workers bear the economic responsibilities of the household, while women are expected to be part of the family household, where they spend a significant amount of physical energy engaging in unpaid family labor, thus becoming dependent on the family income. They are also considered to be “powerless,” that is, at a disadvantage when distributing family resources and assets and lacking decision-making rights on significant family matters. This has resulted in the relative poverty of women’s income, and their dependence on others has led to their relative poverty in the distribution of resources and assets.

Further, in the labor market, considering the gender bias in society and the expected return on investment for employing women, women are often regarded as inferior labor. Under the influence of traditional fixed gender patterns and women’s individual characteristics, women’s access to social capital is worse than men’s and their ability to expand social capital is also limited. They also show substantial homogeneity in their social networks, especially family-centered traditional rural women, which inhibits their individual development. This leads to the relative poverty of women’s social relations and social capital.

Women’s physical characteristics make them more vulnerable than men. For instance, breastfeeding affects their bodies to a certain extent and housework also affects their physical and mental health. Rural women face the influence of traditional cultural values horizontally and are affected by the accumulation of vulnerabilities and risks in different life stages at the vertical level, which leads to the formation of relative poverty among female groups.

Therefore, the formation of the relative poverty of rural women is a multi-dimensional and complex process.

## Materials and methods

### Data sources

The study collected the data from the “Chinese Family Tracking Survey” (CFPS), organized and implemented by the Chinese Social Science Survey Center of Peking University. It mainly traces and collects data from three levels of individuals, families, and communities, with a large sample size. Covering a wide range, including 25 provinces, municipalities, and autonomous regions, it more precisely represents China’s social, economic, population, education, and health conditions.

This study aimed to reflect the long-term multidimensional poverty status of women in the labor force in rural Chinese households and its influencing factors, tracking data from 2010, 2012, 2014, and 2016. The data of 4 years is compared and screened for the adult population of rural households surveyed four consecutive times. The age distribution is between 16 and 60 years old, has a labor force, excludes the school population, obtaining a total population of 9,242, with 4,824 females and 4,418 males.

### Multidimensional poverty dynamic measurement method

This study used the measurement method proposed by Alkire and Foster to measure the poverty of women in rural households. The measurement method is briefly described as follows:

#### Identification of poverty within the dimension

According to the survey database, suppose N represents the adult population of rural households (over 16 years old), D ≥ 2 represents the number of dimensions of the multidimensional poverty measurement, *y* = [*y_ij_*] refers to *N* × *D* the value of the dimensional matrix, *y_ij_* represents the observation value of the individual *i* in the *j* dimension, the row vector *y_i_* is the value of the individual *i*, and the column vector *y_j_* is the value of the *j* dimension, *z_j_* represents the critical poverty value of the dimension, and a deprivation matrix is defined as $g^{0}=g_{ij}^{0}$ , then


(1) $$g_{ij}^{0}=\begin{array}{c} 1\;if\;y_{ij}<z_{j}\\ 0\;if\;y_{ij}\geq z_{j} \end{array}$$

#### Multidimensional poverty identification

The above formula shows the deprivation distribution in each individual dimension. To judge whether the individual faces multi-dimensional poverty, define a column vector to represent the total number of poverty dimensions endured by the individual, that is, sum up the total number of deprived dimensions. Additionally, compare the total number of deprivation dimensions (*c_i_*) with the set multi-dimensional poverty deprivation threshold (k) to determine whether the individual has multi-dimensional poverty. If the number of dimensions an individual is deprived of (*c_i_*) is greater than the set multi-dimensional poverty threshold (k), the individual is deemed to have multi-dimensional poverty; otherwise, there is no multi-dimensional poverty. *ρ_k_* is the function of identifying poverty in k dimensions, *ρ_k_* is affected by both *z_j_* (deprivation within dimensions) and deprivation across dimensions *c_i_*.


(2) $$\rho_{k}\left(y_{i};z\right)=\begin{array}{c} 1\;if\;c_{i}\geq k\\ 0\;if\;c_{i}<k \end{array}$$

#### Multidimensional index

After identifying the deprivation of each dimension, it is necessary to add the dimensions to obtain the comprehensive multidimensional index. *M*_0_ is the adjusted multidimensional poverty index. It consists of two parts: one part is *H* (poverty incidence); the other part is *A* (average deprivation share):


(3) H=H(x,z)=q/n

(4) $$A\left[\overset{n}{\underset{i=1}{\sum }}C_{i}\left(k\right)\right]/\left(qd\right)$$

(5) $$M_{0}\left(x,z\right)=\mu \left(g^{0}\left(k\right)\right)=HA$$

#### The dynamic classification of multidimensional poverty

Based on the multi-dimensional poverty estimation, considering the number of years the rural family population lived in poverty during the tracking year, the poverty dynamics of the tracking population are divided into three types: no poverty, temporary poverty, and chronic poverty. The specific method is as follows: Suppose $p_{k}^{i}$ represents the overall multidimensional poverty dynamics of the i-th individual in T period, $Y_{k}^{i}$ is the poverty year of the i-th individual in T period, and *T*′ is the critical value for judging temporary poverty and chronic poverty. When the i-th individual’s multidimensional poverty year in period T is 0, the poverty status is no poverty; when its multidimensional poverty year is between 0 and *T*′, the status is temporary poverty; when its multidimensional poverty year is greater than *T*′, the status is chronic poverty. The specific division method is as follows:


(6) $$\begin{array}{c} 0\;\;if\;\;Y_{k}^{i}=0\\ p_{k}^{i}=1\;\;if\;\;0<Y_{k}^{i}\leq T^{\prime }\\ 2\;\;if\;\;T^{\prime }<Y_{k}^{i}\leq T \end{array}$$

## Results

### Analysis of multidimensional dynamic measurement results of rural women

#### Dimension selection

This study combined existing research, Oxford Poverty and Human Development Initiative’s UN developmental goals, Human Development Index, and Multidimensional Poverty Index, and based on the availability of CFPS data indicators and the characteristics of adult women in rural households, 15 indexes were selected from six dimensions. The indicator measures the multidimensional poverty of women in rural households. The selection and assignment of each indicator are as follows.

#### Economic dimension

This dimension includes two indicators of personal income and work status. Income level is the most direct indicator to measure the poverty of adult women. The traditional indicator to measure the family’s economic status is the family’s *per capita* income. This indicator often regards the family members as homogeneous and ignores the actuality of hidden income. This study selects personal income and combines it with local comparison to determine the critical value of measuring adult women’s personal income indicators; that is, if the personal income is below the national poverty line and the personal income is relatively low compared to the local, it is assigned a value of 1, otherwise 0. Work status can measure adult women’s employment level and social capital acquisition. Therefore, if adult women have never engaged in work outside of their agricultural activities, the assigned value is 1, otherwise, 0.

#### Health dimension

This dimension primarily includes women’s self-rated health status, the presence or absence of chronic diseases, and body mass index (BMI). Self-evaluation of health is an index that integrates one’s experience and understanding of health in the environment, effectively reflecting individual’s actual health. Therefore, health self-evaluation scores for unhealthy and low-income are assigned a value of 1, otherwise 0. Chronic disease is a measure of an individual’s physical condition at a specific stage and may have long-term effects. The chronic disease within half a year is assigned a value of 1, otherwise 0. The BMI is a standard measure of whether an individual is healthy. Because this article studies low-income families, the BMI is assigned a value of 1 below the minimum standard value, otherwise 0.

#### Human dimension

This dimension includes educational level, information acquisition, and ability to express and understand. It represents the comprehensive quality of adult women. Regarding education level indicators, based on the setting of Multidimensional Poverty Index education indicators, combined with existing data, the value of junior high school and below for adult females is assigned a value of 1, otherwise 0. Information acquisition is how individuals obtain information. It represents the ability of individuals to contact external affairs and use various information channels. The leading information acquisition is through others, does not involve Television, radio, and Internet channels, and is assigned a value of 1, otherwise 0. Expression and comprehension ability is the external manifestation of the humanistic quality of adult women. The CFPS database assigns personal expression and comprehension abilities from low to high as 1–7 through interviewer observation. This study assigned 1 to the index below 3, otherwise, it is assigned 0.

#### Mental life dimension

This dimension includes attention to the news, leisure, entertainment, and life satisfaction indicators. It represents the leisure life and inner satisfaction of adult women and is crucial to measure women’s mental outlook and psychological state. This study assigned a value of 1 to women who never paid attention to any social, political, and similar news; otherwise, the value assigned is 0. If the Internet is never or rarely used, the value assigned is 1; otherwise, 0. Women who are unsatisfied with their lives are assigned a value of 1, otherwise 0.

#### Social dimension

This dimension includes two indicators of personal interpersonal relationships and personal status in the local area. This dimension can measure the social network relationship and social capital acquisition of adult women. The CFPS database assigns the indicators of personal relationships and personal status in the local area from low to high as 0–10 points. In this study, the two indicators are assigned a value of 1 if they are lower than the average value, and otherwise, 0.

#### Rights dimension

This dimension includes two indicators of family decision-making power and property rights. It represents the status of adult women in rural households on life assets and resources and is an important dimension to measure whether the possession of assets and resources is gender-equal. Regarding significant family matters, including important family income and expenditure, property purchases, important activity decisions, and others, the value without decision-making power is 1, otherwise, 0. Rural family property mainly includes real estate, land, among others. According to the available data, the value of 1 is assigned for adult women without real estate rights; otherwise, it is 0.

After determining the indicators of multi-dimensional poverty, determining the weights of the indicators is crucial for calculating the total of multi-dimensional poverty. However, there is no consensus on the multi-dimensional poverty dimension and index weight setting method. This study derives from most domestic and foreign poverty-based research (Xibao and Qiang, 2016). The equal weight method is used in the study of household poverty when there is more than one indicator of any dimension of the multidimensional poverty system. Considering this, this study used the equal-dimensional weight method, assigning the same weight to each dimension, and subsequently, the indicators in each dimension are equally weighted. The dimensions, indicators, and weight settings are shown in Table 1.

**Table 1:** Rural women’s multidimensional poverty dimensions, indicators, deprivation thresholds, and weights.

Dimension	Indicators	Deprivation thresholds	Weights
Economy	Personal income	Assign a value of 1 below the national poverty line, otherwise 0	1/2
	Jobs	Assign a value of 1 for never going out to work, otherwise it is 0	1/2
Health	Physical condition	Unhealthy is assigned a value of 1, otherwise it is 0	1/3
	Chronic diseases	People with chronic diseases within half a year are assigned 1, otherwise 0	1/3
	bmi	Assign a value of 1 if it is lower than 18.5, otherwise it is 0	1/3
Humanities	Education	Education level below junior high school is assigned 1, otherwise 0	1/3
	Access to information	Mainly obtain information through other people’s reports, assign a value of 1, otherwise it is 0	1/3
	Expression and Comprehension	Poor expressive and comprehension ability Assign value 1, otherwise 0	1/3
Spiritual life	News attention	Never pay attention to any social or political news and assign a value of 1, otherwise it is 0	1/3
	Leisure and entertainment	Never or rarely use Internet entertainment to assign a value of 1, otherwise 0	1/3
	Life satisfaction	Dissatisfied with life assign a value of 1, otherwise it is 0	1/3
Socialrelations	Interpersonal relationship	Assign a value of 1 for the difference in personal interpersonal relationship, otherwise 0	1/2
	Personal status	Assign a value of 1 if the individual has a low status in the local society, otherwise it is 0	1/2
Rights	Family decision-making Power	No decision-making power for important family matters is assigned 1, otherwise it is 0	1/2
	Property	There is no assignment of property rights to 1, otherwise it is 0	1/2

### Comparative analysis of poverty incidence rates of rural women and men in various indicators

**Figure 2:**
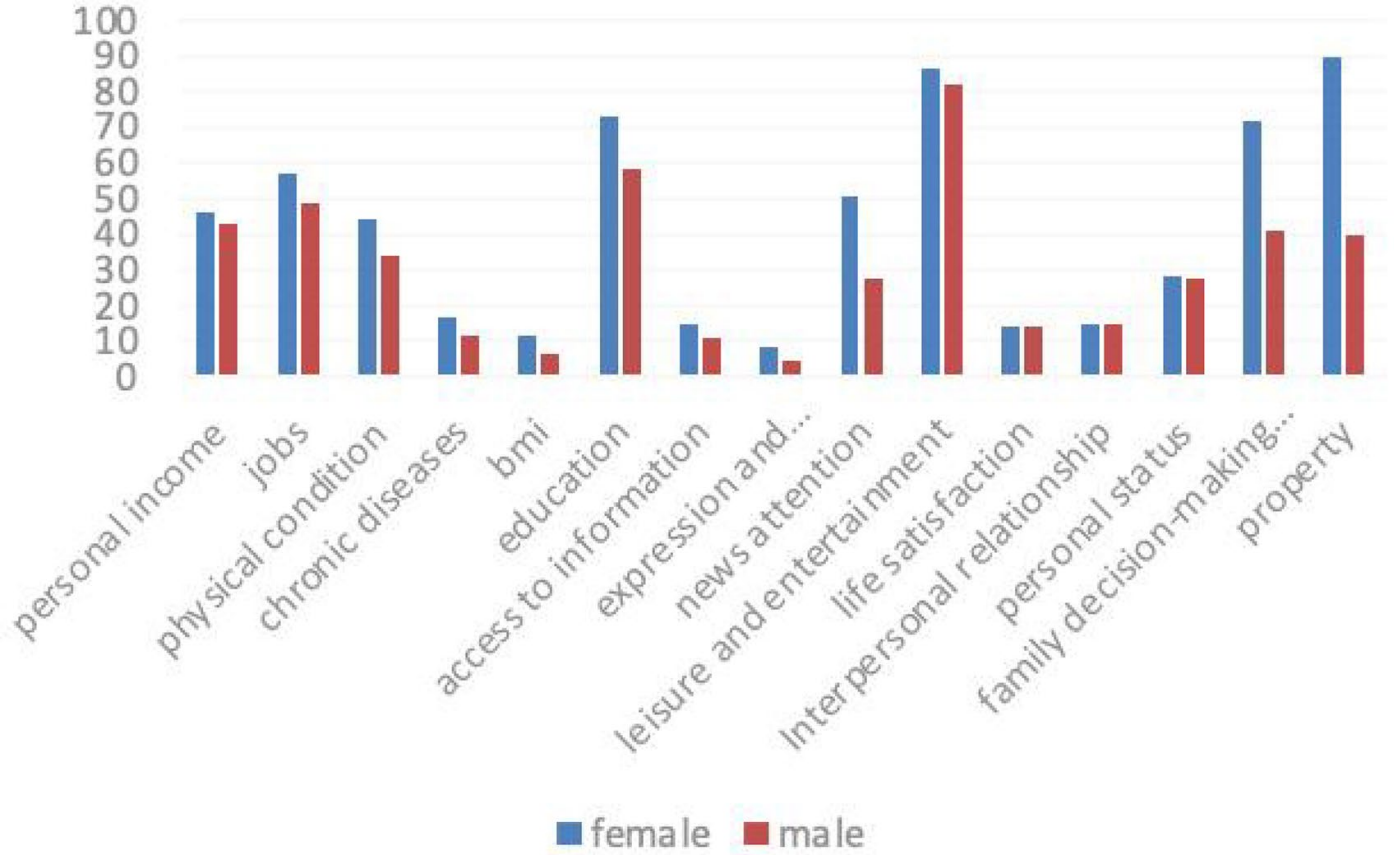
Comparative analysis of poverty incidence rates of rural women and men in various indicators.

As shown in Figure 2, the comparison of the poverty incidence rates of women and men in various indicators indicates that the poverty incidence of women in six dimensions and 15 indicators is higher than that of men, including work status, self-assessed health, education level, and news attention. The gender difference in poverty incidence of household decision-making power and property rights indicators is particularly significant. This shows that women are poorer than men in economy, health, humanities, and rights.

### Comparative analysis of the results of multidimensional poverty measurement of rural women and men

Using the Alkire-foster method, this study measures the multi-dimensional poverty of rural women and compares the measurement results of women’s and men’s multi-dimensional poverty. The results are shown in Table 2. When k = 1–5, the incidence of multidimensional poverty, poverty deprivation share, and multidimensional poverty index of adult women in rural areas are higher than those of adult men. This indicates that the multidimensional poverty level of rural women in China is more severe than that of men.

**Table 2:** Measurement results of multidimensional poverty index for rural women and men.

K	Year	Female				Male			
		Q	H	A	M	Q	H	A	M
K = 1	2010	4,794	99.38%	0.4527	0.4499	3,859	87.35%	0.3389	0.2960
	2012	4,793	99.36%	0.4618	0.4588	3,892	88.18%	0.3370	0.2922
	2014	4,716	97.76%	0.4309	0.4213	4,100	92.80%	0.3619	0.3359
	2016	4,693	97.28%	0.4125	0.4013	3,908	88.45%	0.3320	0.2937
K = 2	2010	3,975	82.40%	0.4921	0.4055	1953	44.21%	0.4339	0.1918
	2012	4,054	84.04%	0.4985	0.4189	1956	44.27%	0.4282	0.1896
	2014	3,790	78.57%	0.4747	0.3729	2,443	55.29%	0.4403	0.2435
	2016	3,499	72.53%	0.4668	0.3386	1868	44.28%	0.4306	0.1821
K = 3	2010	1846	38.27%	0.5919	0.2265	484	10.96%	0.5642	0.0618
	2012	2019	41.85%	0.5894	0.2467	455	10.30%	0.5610	0.0578
	2014	1,518	31.47%	0.5800	0.1825	702	15.89%	0.5611	0.0892
	2016	1,276	26.45%	0.5818	0.1539	445	10.07%	0.5627	0.0567
K = 4	2010	380	7.88%	0.7231	0.0570	51	1.15%	0.7119	0.0082
	2012	399	8.27%	0.7183	0.0594	45	1.02%	0.7006	0.0072
	2014	261	5.41%	0.7059	0.0382	57	0.38%	0.7110	0.0092
	2016	213	4.42%	0.7118	0.0314	40	0.90%	0.7021	0.0063
K = 5	2010	28	0.58%	0.8690	0.0050	5	0.11%	0.8444	0.0009
	2012	22	0.46%	0.8548	0.0039	1	0.02%	0.8611	0.0002
	2014	8	0.17%	0.8507	0.0014	2	0.04%	0.8889	0.0004
	2016	7	0.15%	0.8492	0.0012	1	0.02%	0.8333	0.0002

The different years show that when k = 1, the incidence of multidimensional poverty among rural women is extremely high. The incidence of poverty in 2010–2016 exceeded 95%. This indicates that in these years, most women experienced at least one dimension of poverty. Few women were not poor in all dimensions, and the multidimensional poverty index of rural women was between 0.41 and 0.47. The incidence of multidimensional poverty among men is lower than that of women, and the multidimensional poverty index is between 0.27–0.31. When k = 2, the incidence of poverty in each year decreases, the incidence of poverty among men decreases more than that of women, and the incidence of women’s poverty drops to 85%, the multidimensional poverty index is between 0.33–0.42, and drops below 55% between 0.18–0.25 for men. When k = 3, the female poverty incidence rate drops to below 45%, and the multidimensional poverty index is between 0.15–0.25, while the male poverty incidence rate has dropped below 16% between 0.05 and 0.09 index, indicating an increased number of rural men living for three dimensions is less than 20%, which is approximately 50% for women. When k = 4, the incidence of female poverty drops below 10% and below 5% for men, indicating that women in rural households experience poverty in more than four dimensions compared to men and that the multidimensional poverty index is also small. When k = 5, the poverty incidence rate and multidimensional poverty index for men and women are extremely low, and the poverty incidence rate for women is below 0.6%, while that for men is less than 0.6%. The rate is below 0.2%, indicating a few extremely low-income rural populations experiencing poverty in five dimensions.

To sum up, when k takes different values, both rural women and men show a downward trend in multidimensional poverty, and rural men have a more pronounced decline than women with a significant magnitude. This also confirms that China’s poverty alleviation has had success, but this is often based on rural households as a unit to promote poverty alleviation work, and the premise is that family members are “homogenized,” that is, it is recognized that men and women in low-income families have the same poverty status, while gender differences are not considered. However, families are not homogeneous associations; men and women have certain differences in all aspects, including life, physiology, needs, and welfare. Poverty relief programs that ignore gender differences between men and women contradict the spiritual principle of precise poverty alleviation. Therefore, gender differences must be considered in poverty issues to formulate targeted policies and measures to alleviate poverty among rural women, ensure the accuracy of rural poverty alleviation work, and for a significant role for rural women.

### A comparative analysis of multidimensional dynamic poverty of rural women and men

Furthermore, the type of multi-dimensional poverty is determined following the number of years it was experienced by the rural population. This study utilized 2010–2016 as the period of investigation. If there are 3 years or more of multidimensional poverty in the tracking year, it is judged as chronic poverty; for more than one and less than 3 years in multidimensional poverty, it is judged as temporary poverty; for no poverty during the inspection period, it is judged as not poor.

Table 3 shows the comparison results of multidimensional dynamic poverty between rural women and men. It can be found that when k = 1–5, the proportion of men who have never been poor is larger than that of women, and the proportion of men who have been chronically poor is smaller than that of women. Among them, when k = 2 or 3, the difference is specifically significant. When k = 2, the proportions of women and men who have never been in poverty are 4.19 and 20.21%, respectively. The proportion of men who have never been in poverty is nearly five times that of women. During this time, the proportion of women and men in chronic poverty is 76.68% and 34.56, respectively, nearly twice as high for women as for men. When k = 3, the proportion of men in a state of no poverty is more than twice as high as that of women, and the proportion of women in a state of chronic poverty is higher than that of men. This shows that when k = 2, 3, women in rural households are more in chronic poverty than men, as much as seven times.

**Table 3:** Comparison of the results of multidimensional dynamic measurement of rural women and men.

K	No poverty		Temporary poverty		Chronic poverty	
	Female	Male	Female	Male	Female	Male
1	0.02%	1.00%	0.85%	10.05%	99.13%	88.95%
2	4.19%	20.21%	19.13%	45.22%	76.68%	34.56%
3	33.08%	69.13%	45.11%	26.94%	21.81%	3.94%
4	81.24%	96.36%	17.41%	3.51%	1.35%	0.14%
5	98.69%	99.80%	1.31%	0.20%	0.00%	0.00%

## Discussion

The analyses in section 4.2 and 4.3 indicate that the incidence of poverty among women in the six dimensions of economy, health, humanities, spiritual life, social relations, and rights is higher than that of men. Further, women’s poverty is broader and deeper than that of men. It verifies the findings of the theoretical analysis that the women are relatively poorer than men and their poverty exhibits multiple dimensions. In addition, many aspects, such as, culture, system, self, and environment affect poverty. The continuous infiltration of modern values into the traditional culture results into integration and development of mixed values, which makes the modern society and the people’s decision-making complex.

Keeping in mind the overlapping ideas, this study employs some different terms, such as, “cultural additivity” and “cultural transmitter” to further analyze factors affecting multidimensional relative poverty among rural women. While “cultural additivity” means different cultural value systems and their interactions to influence people’s thinking and behaviors(Vuong et al., 2018), the “cultural transmitter” is the socially acceptable medium for expressing the will and behavior of cultural contradictions (Vuong et al., 2020, p: 82). The poverty of rural women is a complex social system engineering which is influenced by the interaction of factors like history and culture, gender blindness in institutional design, constraints of environmental resources, and economic and social structure. From the perspective of history and culture, traditional Chinese culture possess the deepest and the most ancient foundation for the study of rural women’s poverty. It has continuously matured and evolved to gradually form a view of women that has far-reaching influence on future generations. Traditional social norms, values, and village regulations constitute the value premise of the logical system that affects the relative poverty of rural women, and are internalized into corresponding habits, customs, thinking, and lifestyles. They not only affect the relative poverty but also the all-round development of rural women. The role of rural women’s intergenerational transmission of poverty has a significant impact on the development of the family and the entire society. The gender blindness in institutional design is reflected through the negligence of the protection of women’s interests in the marriage system, land system, market employment, social security, and other systems. The paucity of gender factors in these systems is due to long-term accumulation of intricate and multi-dimensional factors, which had a long-term, profound and subtle effect on the women. The constraints of environmental resources make women more vulnerable for disadvantageous states caused by the integration of various risk factors such as resources, natural disasters, and climate. In the face of the diversification of economic and social structures, women who are in a disadvantaged position because of family resources are often at a loss, and thus more likely to fall into poverty trap.

These factors accompanied by the economic or social developments and changes often form a trap mechanism for rural women to become impoverished. Based on the coupling relationship of mutual connection, influence, and restriction, such traps make rural women more likely to fall into a situation of relative poverty. This problem of rural women’s poverty is not only an ecological or economic problem, but also a political, cultural, and social issue. Therefore, it is necessary to understand the criticality of the matter, and undertake comprehensive, systematic projects to examine it and offer an effective answer to it.

### Variable description

First, the dependent variable selected in this study is the multi-dimensional poverty dynamic type classified above. The analysis shows that when k = 2 or 3, the state of rural women in multidimensional dynamic poverty is significantly different from that of men. Studies typically focus on when the multidimensional poverty k value is greater than or equal to one-third of the total dimension. Therefore, this study selects two situations when the value of k is equal to 2 and 3 to explore the main reasons affecting rural women’s poverty more than men.

Second, this study selected factors affecting rural women’s poverty as independent variables. The selection of these factors mainly includes the characteristics of women’s individual characteristics, families, and villages. Consider the availability of CFPS database data, the selected individual characteristic variables include age, education level, physical condition, whether there are underage children, ideology, and family status. Women in rural families are also responsible for caring for the family and raising children. Therefore, the indicators of whether there are underage children are selected from the personal characteristics of women; women’s ideology can reflect women’s traditional gender cognition and individuality. The consciousness of pursuing self-development is reflected by ideologies of “males focus on careers and females focus on families,” “women are better married,” and “women must have at least one son in order to pass on the family line.” The family status can reflect the living conditions of adult women in the family. This indicator reflects whether women have decision-making power in significant family affairs. Family characteristic variables include family size, labor resources, and durable household goods. The family labor force is between 16 and 60, excluding family members over the age of 16 still in school. Household durable goods are represented by the number of durable household assets. Women’s poverty is also affected by their geographical environment (Xin, 2015); traffic conditions can affect the convenience and quality of life of rural households and have a vital impact on travel for work and medical treatment. Considering this, this study selected the characteristic variables of the village, including medical conditions and traffic conditions. Table 4 shows the overall description results of the independent variables in this study.

**Table 4:** Variable descriptive statistics.

Variable type	Independent variable	Variable description	Mean	SD	Min	Max
Individual characteristics	Age	Age (year old)	45.15	11.68	20	60
	Education	Years of education (years)	2.63	2.12	1	9
	Physical	Health is assigned 1, if not, it is 0	0.64	0.48	0	1
	Child	Assign 1 if there are Children, otherwise 0	0.58	0.49	0	1
	Ideology	With strong traditional Gender awareness are Assigned a value of 1, Otherwise it is 0	0.69	0.46	0	1
	Family status	The higher status is assigned 1, otherwise 0	0.28	0.45	0	1
Family characteristics	Family size	Total family population	4.59	1.90	1	17
	Labor resources	The proportion of family labor force	0.57	0.27	0	3
	Durable good	Number of durable goods (pieces)	0.05	0.16	0	2
Village characteristics	Traffic condition	Proportion of dirt roads in village roads (%)	61.79	36.07	0	100
	Medical	Number of medical clinics and clinics	2.27	2.01	0	14

### Analysis of the dynamic influencing factors of female multidimensional poverty in rural households

Based on the multi-dimensional poverty measurement, this study selected the results of multi-dimensional poverty dynamic decomposition when k = 2 and k = 3 and used the stata14 software using the Probit model to estimate the factors affecting the multi-dimensional poverty dynamics of rural women in rural households from 2010 to 2016. Further, the study analyzed the factors affecting women’s multi-dimensional poverty in rural households and identified which characteristics are more likely to lead to poverty and more capable of reducing the risk of poverty. Table 5 shows the marginal effects of factors affecting the dynamics of multidimensional poverty among rural women when k = 2 and k = 3. The coefficients of each explanatory variable corresponding to no poverty, temporary poverty, and chronic poverty showed opposite signs, and the three arithmetic sum of these coefficients was 0, indicating the probability of rural women falling into various poverty states is opposite.

**Table 5:** The marginal effect of the factors influencing the multidimensional poverty dynamics of rural women.

Variable type	Variable name	K = 2			K = 3		
		No poverty	Temporary	Chronic	No poverty	Temporary	Chronic
Individual characteristics	Age	−0.002^***^	−0.006^***^	0.009^***^	−0.008^***^	0.002^***^	0.006^***^
		(0.000)	(0.000)	(0.000)	(0.000)	(0.000)	(0.000)
	Education	0.007^***^	0.018^***^	−0.025^***^	0.010^***^	−0.002^***^	−0.008^***^
		(0.001)	(0.002)	(0.002)	(0.002)	(0.001)	(0.002)
	Physical	0.037^***^	0.103^***^	−0.140^***^	0.169^***^	−0.033^***^	−0.136^***^
		(0.004)	(0.010)	(0.013)	(0.011)	(0.004)	(0.009)
	Child	−0.013^***^	−0.035^***^	0.048^***^	−0.043^***^	0.008^***^	0.035^***^
		(0.003)	(0.009)	(0.012)	(0.012)	(0.003)	(0.010)
	Ideology	−0.018^***^	−0.050^***^	0.068^***^	−0.037^***^	0.007^***^	0.030^***^
		(0.003)	(0.008)	(0.011)	(0.012)	(0.002)	(0.009)
	Family status	0.022^***^	0.060^***^	−0.082^***^	0.105^***^	−0.020^***^	−0.085^***^
		(0.003)	(0.008)	(0.011)	(0.012)	(0.003)	(0.009)
Family characteristics	Family size	−0.006^***^	−0.016^***^	0.022^***^	−0.023^***^	0.004^***^	0.018^***^
		(0.001)	(0.003)	(0.004)	(0.003)	(0.001)	(0.003)
	Labor resources	0.006	0.018	−0.024	−0.034^*^	0.007^*^	0.028^*^
		(0.006)	(0.016)	(0.022)	(0.020)	(0.004)	(0.016)
	Durable goods	0.008^***^	0.023^***^	−0.031^***^	0.044^***^	−0.009^***^	−0.036^***^
		(0.001)	(0.002)	(0.003)	(0.003)	(0.001)	(0.002)
Village characteristics	Traffic condition	−0.000^***^	−0.001^***^	0.001^***^	−0.001^***^	0.000^***^	0.001^***^
		(0.000)	(0.000)	(0.000)	(0.000)	(0.000)	(0.000)
	Medical	0.003^***^	0.007^***^	−0.010^***^	0.012^***^	−0.002^***^	−0.010^***^
		(0.001)	(0.002)	(0.002)	(0.003)	(0.001)	(0.002)
	Pseudo *R*^2^	0.194			0.126		
	Log likelihood	−2483.989			−4377.579		
	Chi-square	1197.585			1264.229		
	Observations	4,737			4,737		

^***^, ^**^ and ^*^ indicate significant at the statistical level of 1, 5 and 10%, respectively.

Table 5 shows differences in the influencing factors of women’s multidimensional dynamic poverty in different dimensions. When K = 2, the multidimensional dynamic poverty of rural women is mainly affected by education, ideology, and family size. When K = 3, the multidimensional dynamic poverty of rural women is mainly affected by physical conditions, minor children, family status, and durable goods.

From the perspective of individual characteristics, age has a significant positive impact, indicating that as age increases, women are more likely to fall into a state of multi-dimensional dynamic poverty, but since the absolute amount of the coefficient is small, the impact of age on female poverty can be considered minimal. Educational background has a significant negative impact, indicating that the higher the level of education of rural women and the better their overall quality, it will reduce their probability of falling into multi-dimensional dynamic poverty. Physical condition has a significant negative impact, reflecting that the better the physical condition, the less likely it is for women to fall into multidimensional dynamic poverty. Underage children have a significant positive impact on the multidimensional dynamic poverty of rural women, aligning with the actual situation of rural families. Rural women are primarily responsible for child rearing and family care, reducing the space for self-development, and economically family-dependents are often regarded as vulnerable groups and are more likely to fall into poverty. Ideology has a significant positive impact on the multidimensional and dynamic poverty of rural women, indicating that women with strong traditional ideas and gender concepts are more willing to play the role of family women; “men dominate the outside and women dominate the inside” is the persistent outlook to life, creating fear and unwillingness to break the status quo of their lives. Once the family falls into poverty or the marriage is disintegrated, they quickly become poor. Family status has a significant negative impact on rural women’s multi-dimensional dynamic poverty, indicating that the higher the status of women in rural families, the less likely they are to fall into multi-dimensional dynamic poverty. The possible reason is that women’s family status is high, indicating their importance in the family. There is a certain right to participate and make decisions in matters, and they are not “powerless” subjects in the family. They can use various assets and resources at home, helping them resist the risks of multidimensional dynamic poverty.

From the perspective of the impact of family characteristics on rural women’s multidimensional poverty, the impact of family size on women’s multidimensional poverty is significantly positive, indicating that the larger the number of rural families, the easier it is for women to fall into poverty. This may be related to the rural family structure of an intergenerational family, with older adults and underage children in the family. In this family structure, women take more family care responsibilities and are more likely to be poor. The impact of labor resources on the multidimensional poverty of rural families is positive. The possible reason is that the more labor resources in the family, the more prominent the responsibility of women as family caregivers. The number of durable goods in the household is significantly negative for women’s multidimensional poverty. The number of durable household goods is a critical indicator of poverty; the larger the number, the better the economic status of the family. Women from such families are more capable of resisting multidimensional poverty.

From the perspective of the impact of village-level characteristics on rural women’s multidimensional poverty, traffic conditions have a significant positive impact on rural women’s multidimensional dynamic poverty. Women living in an environment with a high proportion of dirt roads are more likely to be poor. This may be because it indicates that the living environment is backward and the infrastructure of the village is inferior, and women living in an environment with backward economic conditions are more likely to fall into multidimensional poverty. Medical conditions have a significant negative impact on rural women’s multidimensional dynamic poverty. Areas with better medical conditions can provide women with more medical and health protection, helping them resist the risks of multidimensional dynamic poverty.

## Conclusions and policy recommendations

This study aimed to construct an analytical framework of rural women’s multidimensional poverty from multiple levels of culture, society, family, and individuals. Using the 2010–2016 CFPS survey data, starting from the “individual perspective,” designed a multidimensional poverty measurement system with six dimensions and 15 indicators and divided the dynamic poverty types according to the duration of individual poverty. Further, the study compares and analyzes the multi-dimensional dynamic poverty of rural women and men and the main factors affecting rural women’s poverty. The main conclusions drawn in this study include the following:

First, regarding poverty incidence in various indicators, in the 2010–2016 tracking years, the average poverty incidence of 15 indicators in the six dimensions of economic, health, cultural, spiritual life, social relations, and rights of rural women was higher than that of men. Among them, there are significant differences in economic, health, humanities, and rights dimensions, confirming that differences in gender education in rural families and the individual women’s characteristics affect women’s income and value in the labor market, and differences in gender income affect their production, decision-making, and the possession of family assets and resources, increasing the possibility of women encountering poverty.

Second, regarding the incidence of multidimensional poverty and its index, a downward trend was observed for rural women and men. However, in the tracking years under different K values, the incidence of women’s multidimensional poverty and the multidimensional poverty index is higher than that of men, indicating that women’s poverty is broader and deeper, confirming that there is a strong “heterogeneity” within rural households.

Third, regarding the dynamic types of multidimensional poverty, rural women and men are in a state of multidimensional dynamic poverty. Women in rural areas are in a state of chronic poverty more often than men, indicating that women’s poverty lasts longer than men’s, and the multidimensional dynamics of poverty are more pronounced than that of men. This shows that more attention must be given to women’s poverty.

Fourth, regarding the main factors affecting rural women’s multidimensional dynamic poverty, individual characteristics and family characteristics have a significant impact. A higher level of education, good health, and a high family status can significantly reduce women’s involvement in multidimensional poverty. In contrast, minor children, families with strong traditional ideologies, and large-scale structures can easily cause women to fall into multidimensional poverty.

### Policy recommendations

This study has the following recommendations.

First, change social ideology and attach importance to gender equality. In acknowledging gender differences, society should weaken the gender role, attach importance to the role of rural women in production and life, economic development, and social construction, strengthen investment in rural women’s human capital, enhance women’s social rights status, and protect rural women from having equal access to social benefits such as education, medical care, and social security, explore their potential for participating in social, economic, and cultural construction, and encourage and support them to participate in various social and economic activities for rural revitalization.

Second, attention must be paid to the heterogeneity within rural households to reduce gender poverty gaps. The current targeted poverty alleviation programs have achieved precision for the “household”; however, the substantial heterogeneity within rural households determines that women are more vulnerable to poverty. Therefore, poverty alleviation work requires the accuracy of the “household” and also the “person.” The government should target the needs of women and formulate targeted poverty alleviation policies. Government departments should strengthen publicity and guidance, increase investment in human capital for women in rural families, enhance society’s awareness of rural women’s contributions to all aspects of family production and life, enhance their status as the main body of the family, and earnestly safeguard and protect their legitimate rights and interests.

Third, stimulating rural women’s self-development awareness and improving their comprehensive quality capabilities are needed. Rural women’s weak self-development awareness and low comprehensive quality capabilities are the current resources influencing factors for rural women’s low family status and poor personality independence. Therefore, the government must strengthen cultural education, vocational training, employment guidance, labor protection, and rights protection for rural women. Simultaneously, combined with the rural revitalization strategy, vigorously performing rural cultural revitalization and effectively transforming the backward traditional cultural customs in rural areas for the development of women, thoroughly stimulating and releasing their self-reliance, self-esteem, and self-improvement consciousness of self-development is needed.

This study has some limitations. First, some variables might have been overlooked. Because of the limited issues of the CFPS data, we did not control for environmental factors that affect the relative poverty of rural women. For example, factors such as population mobility, urbanization rate, and digital economy may have an impact on the relative poverty of rural women, however, these information are missing in the CFPS data, making it impossible to examine the effect of these factors on the relative poverty of rural women. Second, in this study, we studied the relative poverty of adult women in rural households. However, there are some elderly women among adult women, the maintenance and other issues, should also be included. Unfortunately, due to limitations related to the questionnaire survey data, it was not possible to conduct more detailed analyses.

## Data availability statement

The datasets presented in this study can be found in online repositories. The names of the repository/repositories and accession number(s) can be found at: http://www.isss.pku.edu.cn/cfps/.

## Ethics statement

Ethical review and approval were not required for the study on human participants in accordance with the local legislation and institutional requirements. Written informed consent from the [patients/ participants OR patients/participants legal guardian/next of kin] was not required to participate in this study in accordance with the national legislation and the institutional requirements.

## Author contributions

The author confirms being the sole contributor to this work and has approved it for publication.

## Funding

This study received the following grants: Philosophy and Social Science Research Project of Hubei Provincial Department of Education, “Research on the identification of stable poverty alleviation and the prevention and control management mechanism of returning to poverty in the old revolutionary base area: Taking the old area of Dabie Mountain in Hubei as an example” (203202135503); Guidance Project of Scientific Research Program of Hubei Provincial Department of Education, “Research on the Development Status and Implementation Path of Digital Agriculture in Hubei Province” (B2021241); Ph.D. Fund Project “Construction of Support System for Rural Industry: Revitalization and Women’s Industry Project Participation” (2042021027) and College Students' Innovative Entrepreneurial Training Project “Is there a "digital dividend" for female entrepreneurs in rural areas? -- Based on the analysis of survey data in Hubei Province” (grant number:2022dc038).

## Conflict of interest

The author declares that the research was conducted in the absence of any commercial or financial relationships that could be construed as a potential conflict of interest.

## Publisher’s note

All claims expressed in this article are solely those of the authors and do not necessarily represent those of their affiliated organizations, or those of the publisher, the editors and the reviewers. Any product that may be evaluated in this article, or claim that may be made by its manufacturer, is not guaranteed or endorsed by the publisher.
